# 

**Published:** 1893-02-18

**Authors:** 


**Notices of Births, Deaths, and Marriages, are inserted in this column at a charge of 2s. 6d. for an announcement not exceeding four lines.**

### NOTICE TO CORRESPONDENTS.

All MS., Letters, Books for Review, and other matters intended for the Editor should be addressed The Editor, The Lodge, Porchester Square, London, W.

The Editor cannot undertake to return rejected M.S., even when accompanied by stamped directed envelope.

**Notice to Advertisers and Subscribers.**

All Advertisements, Orders for Copies of The Hospital, and Business Communications should be addressed to The Publisher, *not to the Editor*, The Hospital, 140, Strand, W.C.

The Cost of Subscription, post free, is as follows:—Per week, 2½d.; per quarter, 2s. 6d.; per half-year, 5s.; per annum, 8s. 6d. Foreign:—Per annum, 10s. 8d.; payable, in all cases, in advance.

**"Burdett's Hospital Annual," 1893.**

Fourth year of publication. 700 pp. Boards, gilt lettering. A most complete guide to British, Colonial, Indian, and American Hospitals, Dispensaries, Nursing and Convalescent Institutions, and Asylums. Price 5s. Published by The Scientific Press (Limited), 140, Strand, W.C.

**NOTICE.—The Index for Vol. XII. (ending September 1892) is on sale at the Office of this Journal, price 2½d., post free.**

### SCRAPS AND GLEANINGS.

In Berlin an act is in force rendering hotel keepers responsible for the cleanliness of all drinking vessels used by them.

It has been decided to extend the operations in connection with the coal boring at the Channel Tunnel works near Dover.

The annual meeting of the Cancer Hospital, Brompton, will be held on Wednesday, 22nd inst., at the hospital at four p.m.

The fire which occurred recently at the Newcastle Literary and Philosophical Institution is estimated at a loss of £20,000.

The Government of Japan has repealed the law hitherto existing, which enforced the marriage of women after a proscribed age.

Sir Algernon Borthwick, M.P., will preside at the annual general meeting of the Newsvendors' Benevolent and Provident Institution, to be held at the Memorial Hall, Farringdon Street, 23th February.

Over 300 buildings, hotels, business houses, and private residences have been erected at Jerusalem since the railway has been run to the sacred city from Jaffa.

During last month 3,431 aliens arrived from the Continent at ports in the United Kingdom, of whom 1,830 were stated to be on their way to America.

Fischer-Peter, of Worms, has just brought out a new filter, which requires little attention and more effectually clears the water than any other in use.

The Home Teaching Society for the Blind of London, has received a legacy of £300, by the will of the late Miss Frances Smith, of Downs Road Epsom.

In Denmark it is the law that all drunken persons shall be taken to their homes in carriages provided at the expense of the publican who sold the last glass.

About 1,400 million gallons of milk are produced in the United Kingdom every year, of which quantity 400 millions are used in the production of butter and cheese.

The Royal Agricultural Show for 1894, which will be held at Cambridge, will, it is expected, take place in Midsummer Common, and the site will consist of 70 acres.

The Duke of Westminster, in making a general appeal on behalf of the interests of the London Temperance Hospital, has given £[illegible]00 to that deserving institution

The finances of the Devon and Exeter Hospital are not in a very flourishing condition; an organised effort is to be made to increase the funds of the establishment.

The proposal to erect a fever hospital in Epping Forest has been met with unanimous objections from the ratepayers of Woodford, who assert that the Forest should be retained as a recreation ground alone.

Mr. C. H. Gore, Vice-Principal of the Liverpool College, has received the appointment of headmaster of Hymer's College, at Hull, which has been built and endowed under the terms of the late Dr. Hymer's will.

The "Concordia" Society of Paris is promoting the use of a gas kitchen-oven for work-people. This is so arranged that no fumes or gas can escape, and it will heat the room and cook the food at the same time.

One of the most interesting spots in Old Kensington is about to be swept away. The builder is soon to demolish the "Terrace" where John Leech lived, and whither all the wits and litterateurs of his day flocked.

A memorial has been presented to the Necropolis Committee in Glasgow from a Cremation Society asking for permission to build a crematorium and columbrarium for the deposit of cinerary urns in the Necropolis.

In consequence of the small-pox epidemic at Lanchester, a motion was passed at the monthly meeting of the Lanchester Joint Hospital to the effect that one ward should be reserved for small-pox patients in that vicinity.

We hear of a new instrument of torture of American design and manufacture. This is a corset for the foot! Thus enabling the wearer to use one size smaller in shoes, than nature, untampered with, would admit of.

There is a new invalid food, which is highly recommended by Dr. Noerdlinger for its nourishing properties. It is prepared from the flour of a nut—the fruit of the Arachis-hypogœa. This food has a pleasant flavour, and keeps well.

The Austrian Government has invited Great Britain to send delegates to an international conference to meet shortly at Dresden, for the consideration of a universal understanding between nations for the prevention of the spread of cholera.

The report of the Brighton Hospital for Women says that, towards the £800 required for the rebuilding of the out-patients' department, small sums only had so far been received. The necessity for the provision of the accommodation is most urgent.

The Johns Hopkins Hospital Bulletin has lately received some observations on bacteria found in the interior of large hailstones. The majority of organisms, which ranged from 400—700 to the cubic centimetre represented only a single species, a short, thin bacillus.

Mr. R. L. Garner, who is continuing his studies of the speech of the gorilla and chimpanzee in Africa, purposes applying electricity for his researches. Telephones are to be run to the gathering places of the apes, and connected to phonographs in the cage where the researcher will reside.

In accordance with the recommendation of the committee of the Glasgow Royal Infirmary, the wards of one of the physicians and one of the surgeon's have been alloted for the separate clinical instruction of female students, while separate pathological instruction is also afforded to them.

The Committee of the Dental Hospital of London are raising funds for the acquisition of a new site in Leicester Square, the sanitation and accommodation of the old establishment being inadequate. H.R.H. the Duke of Cambridge, the President, cordially supports the idea of the proposed new hospital, and H.R.H. the Prince of Wales has sent a donation towards the £40,000 required.

For Student of Medicine and Medical Appointments see pages VIII. and IX. overleaf.

### THE STUDENT OF MEDICINE.

[All communications for this Supplement should be addressed to THE EDITOR of THE HOSPITAL, at 140, Strand, with the words, "Students' Column," written in the left hand top corner of the envelope.]

### NOTES FROM THE SCHOOLS.

### Westminster Hospital.

RETIREMENT OF THE LIBRARIAN.

On Friday the 4th inst., at the Medical School, Caxton Street, before a large gathering, Mr. John Green, the popular and esteemed Secretary and Librarian, was presented with a testimonial and purse containing over a hundred pounds. Dr. Donkin, senior physician to the hospital and lecturer on medicine, on making the presentation, referred in most touching terms upon the compulsory retirement, due to a form of paralysis, of Mr. Green; his good qualities and untiring devotion to the school and all connected therewith. In finishing he added that the good wishes of all were extended with the purse and testimonial. Dr. Donkin then called upon Mr. Nitch-Smith to read the testimonial, of which the following is a copy :—

"Westminster Hospital Medical School, 3rd Feb., 1893.

"Dear Mr. Green,—While expressing to you our warm sympathy in the distressing circumstances under which you are retiring from our midst, we hope that a period of rest and quiet will enable you to return to us in health and strength. Until we tried to find for our feelings towards you words that might suitably accompany the token we are asking you to accept, we hardly realised how much the school as an institution, and we personally, owe to your loyalty and devotion. To all of us, to the earnest and the idle alike, in our best and in our worst moments you have ever been a true and kindly friend, a faithful and gentle monitor, always unweariedly forwarding every worthy thing we have attempted. And even in our sorrow at parting from you we feel that your influence will remain and help us to fulfil a higher purpose in life and devote a more unselfish love to our profession.

"Committee representing the Westminster Hospital Medical School.

G. COWELL.

H. B. DONKIN.

M. FARRANT.

H. J. FINCH.

H. H. MILLS.

R. NITCH-SMITH."

In acknowledging the presentation of the testimonial and purse, Mr. Green expressed his deep sense of the honour that had been done him by the gathering of so many members of the school, staff, and students on that occasion. He dwelt upon the cordial relations which had always existed between himself and the students, and thankfully recognised the support he had received from the school authorities. Although he could only count six years' service, they had been the happiest and most fruitful of his life, and he very keenly felt the severance of the many ties which had bound him to the school. He had no higher ambition than to spend his life in such congenial service. In conclusion, might success attend their efforts towards advancement in their profession, and might they always cherish kindly thoughts for their old hospital. Mr. Green then sat down midst loud and continued applause. An impressive ceremony was brought to a close after a vote of thanks was passed to the chairman, Dr. Donkin.

### PASS LIST.

**Royal Colleges of Physicians and Surgeons of Edinburgh, and Faculty of Physicians and Surgeons of Glasgow.**

The quarterly examinations in Edinburgh for the triple qualification took place in January, with the following results :—

FIRST EXAMINATION.—Of 43 candidates, the following 30 passed: C. A. Francois, Mauritius; H. Bateson, Bolton; E. P. Mead, Whitby; C.

### MEDICAL APPOINTMENTS.

THE HOSPITAL FOR SICK CHILDREN,
Great Ormond Street, Bloomsbury, W.C.

### THE STUDENT OF MEDICINE—(*continued*).

Delacherois, Donaghadee ; J. N. Williams, Brecon ; J. M. Adam, Liverpool ; J. Johnson, Aspull ; A. Kennedy, Ayr ; H. M. McL. Mackenzie, Whitehaven ; A. Speight, Adlington ; C. H. B. Adams, Birmingham ; T. B. Charlton, Blyth ; P. H. Hadfield, Birkenhead ; F. H. Wolfe, Glasgow ; F. S. Gregory, Edinburgh ; H. Macfarlane, Balfron ; J. Hartley, Accrington ; H. J. Fern, Glasgow ; J. Moore, county Antrim ; A. Bance, Madras ; T. W. Mason, Glasgow ; T. Duff, Duddingston ; M. P. O'Shaughnessy, county Kildare ; R. Finlay, Linlithgowshire ; O. Johnson, Lincolnshire ; H. Downes, Devonshire ; H. Holden, Lancashire ; Mary Stuart Sinclair Coghill, Shanghae ; S. Johnson, Manchester ; and Elizabeth Henderson, London. Of 11 candidates who entered for the respective divisions, five passed. *Under Five Years' Course.*—The following candidates passed the entire examination: Mary Ariel Stewart, Portishead ; and Euphemia Cumming, Edinburgh. Twelve candidates entered for the respective divisions and passed.

### VACANCIES.

The following vacancies are announced this week, the amount of salary in each case being given after the name of the institution :

### Resident Medical Officers.

Buckinghamshire General Infirmary, Aylesbury. Resident Surgeon and Apothecary. Unmarried. Doubly qualified. Salary £80 for the first year, rising £10 annually to £100, with board, washing, coals, and candles, unfurnished apartments. Applications to Mr. George Fell, Solicitor, Aylesbury, by February 27th.

Chichester Infirmary, Chichester. House Surgeon. Salary £80 per annum, with board, lodging, and washing. Applications to Mr. E. E. Street, Secretary, by March 1st.

Children's Hospital, Steelhouse Lane, Birmingham. Assistant Resident Medical Officer. Salary £40 per annum, with board, washing, and attendance in the institution. Applications to the Secretary by February 22nd.

General Hospital, Birmingham. House Physician. Salary £70 per annum, with board and lodging. Applications by February 25th to the House Governor.

London Temperance Hospital, Hampstead Road, N.W. House Surgeon. Doubly Qualified. Appointment for six months. Salary at the rate of 50 guineas per annum, with residence in the hospital, board and washing. Applications to E. Wilson Taylor, Secretary, by March 3rd.

London Temperance Hospital, Hampstead Road, N.W. Junior House Surgeon. Appointment for six months. Residence in the hospital, board and washing provided, and an honorarium of 5 guineas at satisfactory completion of term of appointment. Applications to E. Wilson Taylor, Secretary, by March 3rd.

North Riding Asylum, Clifton, York. Temporary Assistant Medical Officer. Salary 2 guineas a week, with board, lodging, washing, and attendance. Applications to the Medical Superintendent.

Oldham Infirmary. Senior House Surgeon. Doubly qualified. Salary £80 per annum, with board, lodging, and washing. Applications to the Secretary, by February 20th.

St. Andrew's Hospital for Mental Diseases, Northampton. Assistant Medical Officer. Doubly qualified. Unmarried. Salary to commence at £200 per annum, rising £25 a year to £250, with board, lodging, and washing. Applications to the Medical Superintendent by February 20th.

St. Mark's Hospital for Fistula and other Diseases of the Rectum, City Road, E.C. Anæsthetist. Honorarium £50 per annum. Applications to the Secretary by February 25th.

### Non-Resident Medical Officers.

Hospital for Sick Children, Great Ormond Street, Bloomsbury, W.C. Surgeon. Must be F.R.C.S.Eng. Applications to the Secretary by February 21st.

Hospital for Sick Children, Great Ormond Street, Bloomsbury, W.C. Assistant Surgeon. Must be F.R.C.S.Eng. Applications to the Secretary, by February 21st.

Mason College, Birmingham. Demonstrator in Physiological Department. Applications to G. H. Morley, Secretary, by February 24th.

Mason College, Birmingham. Professor of Gynæcology. Applications to G. H. Morley, Secretary, by February 24th.

Parish of Glenelg. Medical Officer for the Northern Division. Salary £95 per annum, with free house and garden. Applications to D. M. Lure, Inspector of the Poor, Glenelg, by February 21st.

Royal College of Surgeons of England. Examiner in Elementary Biology. Applications to the Secretary by February 23rd.

